# Nicotinamide and WLD^S^ Act Together to Prevent Neurodegeneration in Glaucoma

**DOI:** 10.3389/fnins.2017.00232

**Published:** 2017-04-25

**Authors:** Pete A. Williams, Jeffrey M. Harder, Nicole E. Foxworth, Brynn H. Cardozo, Kelly E. Cochran, Simon W. M. John

**Affiliations:** ^1^The Jackson Laboratory, Howard Hughes Medical Institute Bar Harbor, ME, USA; ^2^Department of Ophthalmology, Tufts University of Medicine Boston, MA, USA

**Keywords:** glaucoma, NAD^+^, *Wld* ^*S*^, axon degeneration, retinal ganglion cell

## Abstract

Glaucoma is a complex neurodegenerative disease characterized by progressive visual dysfunction leading to vision loss. Retinal ganglion cells are the primary affected neuronal population, with a critical insult damaging their axons in the optic nerve head. This insult is typically secondary to harmfully high levels of intraocular pressure (IOP). We have previously determined that early mitochondrial abnormalities within retinal ganglion cells lead to neuronal dysfunction, with age-related declines in NAD (NAD^+^ and NADH) rendering retinal ganglion cell mitochondria vulnerable to IOP-dependent stresses. The Wallerian degeneration slow allele, *Wld*^*S*^, decreases the vulnerability of retinal ganglion cells in eyes with elevated IOP, but the exact mechanism(s) of protection from glaucoma are not determined. Here, we demonstrate that *Wld*^*S*^ increases retinal NAD levels. Coupled with nicotinamide administration (an NAD precursor), it robustly protects from glaucomatous neurodegeneration in a mouse model of glaucoma (94% of eyes having no glaucoma, more than *Wld*^*S*^ or nicotinamide alone). Importantly, nicotinamide and *Wld*^*S*^ protect somal, synaptic, and axonal compartments, prevent loss of anterograde axoplasmic transport, and protect from visual dysfunction as assessed by pattern electroretinogram. Boosting NAD production generally benefits major compartments of retinal ganglion cells, and may be of value in other complex, age-related, axonopathies where multiple neuronal compartments are ultimately affected.

## Introduction

Glaucoma is complex, multifactorial disease characterized by the progressive dysfunction and loss of retinal ganglion cells. Affecting 80 million people, glaucoma is a leading cause of vision loss worldwide (Quigley and Broman, 2006) and is one of the most common neurodegenerations. Major risk factors include increased intraocular pressure (IOP) and age. Degeneration of the retinal ganglion cell during glaucoma is a compartmentalized process, with differing mechanisms damaging distinct compartments (soma, axon, dendrite, and synapse; Whitmore et al., 2005). Genetic or pharmacological targeting of processes underlying compartmental damage only provides partial protection against glaucoma [e.g., inhibition of somal degeneration through genetic removal of *Bax* (Libby et al., 2005b) and preventing synapse elimination through the genetic or pharmacological inhibition of C1 (Williams et al., 2016)]. It is important to discover strategies that protect all neuronal cell compartments as this may allow additional cell survival and increased neuronal function. Therapies that target early dysfunction and protect multiple neuronal compartments are likely to be the most effective at preserving vision.

The DBA/2J (D2) mouse is a widely used model of glaucoma, which recapitulates the hallmark features of the human disease (Libby et al., 2005a; Nickells et al., 2012). In D2 mice in our colony, ocular hypertension occurs in the majority of eyes by 8–9 months of age (mo). Retinal ganglion cells are damaged as early as 9 mo with disrupted electrical activity (Porciatti, 2015), and dendritic and synaptic atrophy without detectable RGC loss or optic nerve degeneration (Williams et al., 2013, 2016). Optic nerve degeneration occurs from 10 mo onwards with local axonal dystrophy being an early event. To understand the earliest molecular changes that occur in glaucoma, we have used RNA-sequencing (RNA-seq) to analyze D2 retinal ganglion cells at different ages. This identified mitochondrial dysfunction and metabolite depletion (most notably reduction in total NAD [NAD(t); NAD^+^ and NADH)] as a primary driver of early damage in glaucoma. Restoration of NAD, using a diet supplemented in nicotinamide (NAM; an NAD precursor), or through gene therapy (over expression of *Nmnat1*; coding an NAD producing enzyme), protects from glaucoma (Williams et al., 2017). Based on the presence of early glaucomatous damage in axons and synapses we hypothesize that targeting NAD-based treatments to these neuronal compartments will prove to be an even more effective treatment.

The Wallerian degeneration slow allele (*Wld*^*S*^) is a chimera of *Ube4b* and *Nmnat1* producing a fusion protein, WLD^S^ (Mack et al., 2001). WLD^S^ protects from an array of neurodegenerations including glaucoma (Howell et al., 2007; Beirowski et al., 2008). WLD^S^ has NAD producing activity (Wang et al., 2005; Coleman and Freeman, 2010; Wu et al., 2011), and improves mitochondrial function and viability after injury (Avery et al., 2012; O'Donnell et al., 2013). Thus, WLD^S^ provides a promising option for combinational therapy with NAM.

In the present study, we show that WLD^S^ has a role in NAD(t) production in the retina, that both WLD^S^ and NAM protect synapses and axons in glaucoma, and that NAM supplementation further reduces the risk of severe glaucomatous optic nerve degeneration in *Wld*^*S*^ D2 eyes. Remarkably, this combination of enzyme and precursor is profoundly protective and prevents glaucoma in 94% of eyes. This combination may be of substantial benefit in other neurodegenerative models, especially where WLD^S^ alone may be less effective due to age or other causes of NAD depletion.

## Methods

### Mouse strains, breeding, and husbandry

Mice were housed and fed in a 14 h light/10 h dark cycle with food and water available *ad libitum*. All breeding and experimental procedures were undertaken in accordance with the Association for research for Vision and Ophthalmology Statement for the Use of Animals in Ophthalmic Research. The Institutional Biosafety Committee and the Animal Care and Use Committee at The Jackson Laboratory approved this study. DBA/2J (D2), DBA/2J-*Gpnmb*^*R150X*^ (D2-*Gpnmb*^+^), and DBA/2J.Bola-*Wld*^*S*^/Sj (D2.*Wld*^*S*^) strains were utilized and have been described in detail elsewhere (Libby et al., 2005a; Howell et al., 2007). D2-*Gpnmb*^+^ mice do not develop high IOP or glaucomatous neurodegeneration. For aged glaucoma experiments D2 or D2.*Wld*^*S*^ mice were administered NAM in food and/or water starting at 6 mo (prophylactic, prior to high IOP). NAM (550 mg/kg/d; PanReac AppliChem) was dissolved in regular acid drinking water (350 ml) and changed once per week.

### Differential gene expression and pathway analysis

RNA-sequencing datasets were utilized from publically available RNA-sequencing transcripts generated by our lab and published previously (Gene Expression Omnibus accession number GSE90654). Adjustment for multiple testing was performed using false discovery rate (FDR, *q*). Genes were considered to be significantly differentially expression at an FDR < 0.05. For pathway analysis, QIAGEN's Ingenuity Pathway Analysis (IPA, Qiagen) was used for network generation across genes that are significantly differentially expressed.

### NAD^+^/NADH quantification

For NAD^+^/NADH [NAD total; NAD(t)] quantification retinas were dissociated as previously described and measured following the manufacturer's instructions (Biovision). Results were calculated according to the standard curve generated by using standards from the kits. Final NAD(t) concentrations for each samples were normalized to total protein concentration measured by DC assay.

### Clinical phenotyping

In all experiments, the progression of the iris disease and IOP in mutant or drug-treated mice were compared to control D2 mice as previously described (John et al., 1998; Chang et al., 1999). Briefly, for IOP measurement, mice were anesthetized and securely placed on a surgical platform. A drop of PBS was placed on the cornea to prevent dehydration and the anterior chamber was cannulated using a pulled glass microneedle attached to a micromanipulator. IOP was recorded as outlined in John et al. (1997) and Savinova et al. (2001). In each experiment, iris disease and IOP were assessed. Iris disease was assessed at 2-month intervals starting at 6 months of age until experiment completion. Intraocular pressure was measured at 45-day intervals beginning at 8–9 mo until experiment completion.

### Pattern electroretinography (PERG)

PERG was recorded subcutaneously from the snout as previously reported (Chou et al., 2014). Briefly, patterned stimuli (gratings of 0.05 cycles/degree, 100% contrast) generated on LED panels were presented at each eye separately with slight different frequencies around 1 Hz. Waveforms were retrieved using an asynchronous averaging method. Mice were anesthetized using ketamine/xylazine (Savinova et al., 2001) and body temperature maintained at 37°C on a feedback-controlled heated stage monitored by rectal thermometer.

### Optic nerve assessment and grading of glaucomatous damage

The processing of optic nerves and staining with paraphenylenediamine (PPD) was as previously reported (Smith et al., 2002). PPD stains the myelin sheath of all axons but darkly stains the axoplasm of only damaged axons. It is well-established to provide a very sensitive measure of optic nerve damage. Briefly, intracranial portions of optic nerves were fixed in 4% PFA at RT for 48 h, processed and embedded in plastic. A segment of optic nerve from within a region up to 1 mm from the posterior surface of the sclera was sectioned (1 μm thick sections) and stained with PPD. Typically 30–50 sections are taken from each nerve. Multiple sections of each nerve were considered when determining damage level. Optic nerves were analyzed and determined to have one of three damage levels:

1. No or early damage (NOE)—Less than 5% axons damaged and no gliosis. This level of damage is seen in age and sex matched non-glaucomatous mice and is not due to glaucoma. Although none of these eyes exhibit glaucomatous nerve damage, this damage level is called no or early glaucoma as some of these eyes have early molecular changes that precede neurodegeneration (Howell et al., 2011). These molecular changes can be detected by gene expression studies. Eyes with these early molecular changes but no degeneration are considered to have early glaucoma when discussing metabolic, mitochondrial and gene expression changes in this paper.
2. Moderate damage (MOD)—average of 30% axon loss and early gliosis,
3. Severe (SEV)—Greater than 50% axonal loss and damage with prominent gliosis.

### Anterograde axon transport

Mice were anesthetized using ketamine/xylazine and intravitreally injected with 2 μl AF488 cholera toxin subunit B (1 mg/ml in PBS; ThermoFisher Scientific). After 72 h mice were anesthetized and euthanized via 4% PFA cardiac perfusion. Brains and eyes were post-fixed in 4% PFA for an additional 24 h, cryoprotected in 30% sucrose in PBS overnight (ON), OCT cryoembedded, and sectioned at 20 μm. AF488 was visualized using a Zeiss AxioObserver or Zeiss AxioImager.

### Histology

For immunofluorescence staining, mice were euthanized, their eyes enucleated, and placed in 4% PFA overnight (ON). Retinas were dissected and flatmounted onto slides, permeabilized with 0.1% Triton-X for 15 mins, blocked with 2% BSA in PBS, and stained ON at RT in primary antibody. After primary antibody incubation, retinas were washed five times in PBS, stained for 4 h at RT with secondary antibody. Antibodies used are shown in Table 1. Slides were then washed a further five times with PBS, stained with DAPI for 15 mins, mounted with fluoromount, coverslipped, and sealed with nail-polish. For retinal sections, eyes were cryoprotected in 30% sucrose ON, frozen in OCT, and cryosectioned at 18 μm. Slides were warmed to room temperature and the procedure above was followed. For immunofluorescence quantification Z-stack images were Z-projected, the color channel with the secondary fluorophore cropped out, a region of interest placed around the IPL, and mean pixel intensity measured (as in Bosco et al., 2011; Williams et al., 2012, 2016, 2017; Samuel et al., 2014). Retinas were imaged using a Zeiss AxioObserver in one session with exposure limits set the same for all images. For Nissl staining frozen sections were warmed to room temperature, placed in 1:1 alcohol:chloroform ON, and rehydrated through serial alcohol gradient. Slides were washed once in distilled water and stained for 15 mins in 0.1% cresyl violet in distilled water before being differentiated in 95% alcohol, dehydrated in 100% alcohol, and cleared in xylene. Slides prepared as above. Nissl stained retinal sections were imaged using a Nikon Eclipse E200. All images were prepared using FIJI (ImageJ).

**Table 1:** **Antibodies used in this study**.

**Antibody**	**Concentration**	**Manufacturer**	**Catalog number**
GAP-43	1:500	Abcam	Ab16053
RBPMS	1:500	Novus Biologicals	NBP2–20112
Synaptophysin	1:250	Synaptic Systems	101002
TOM20	1:250	Abcam	Ab78547

### Statistical analysis

The sample size (number of eyes, *n*) is shown in each figure legend. Graphing and statistical analysis was performed in R. *Student's t*-test was used for pairwise analysis in quantitative plots, *Fisher's exact* test was used for nerve grade comparisons. Error bars refer to standard error of the mean unless otherwise stated. ^*^P < 0.05, ^**^P < 0.01, ^***^P < 0.001.

## Results

### RNA-sequencing implicates early axonal and synaptic dysfunction in glaucoma

**Figure 1:**
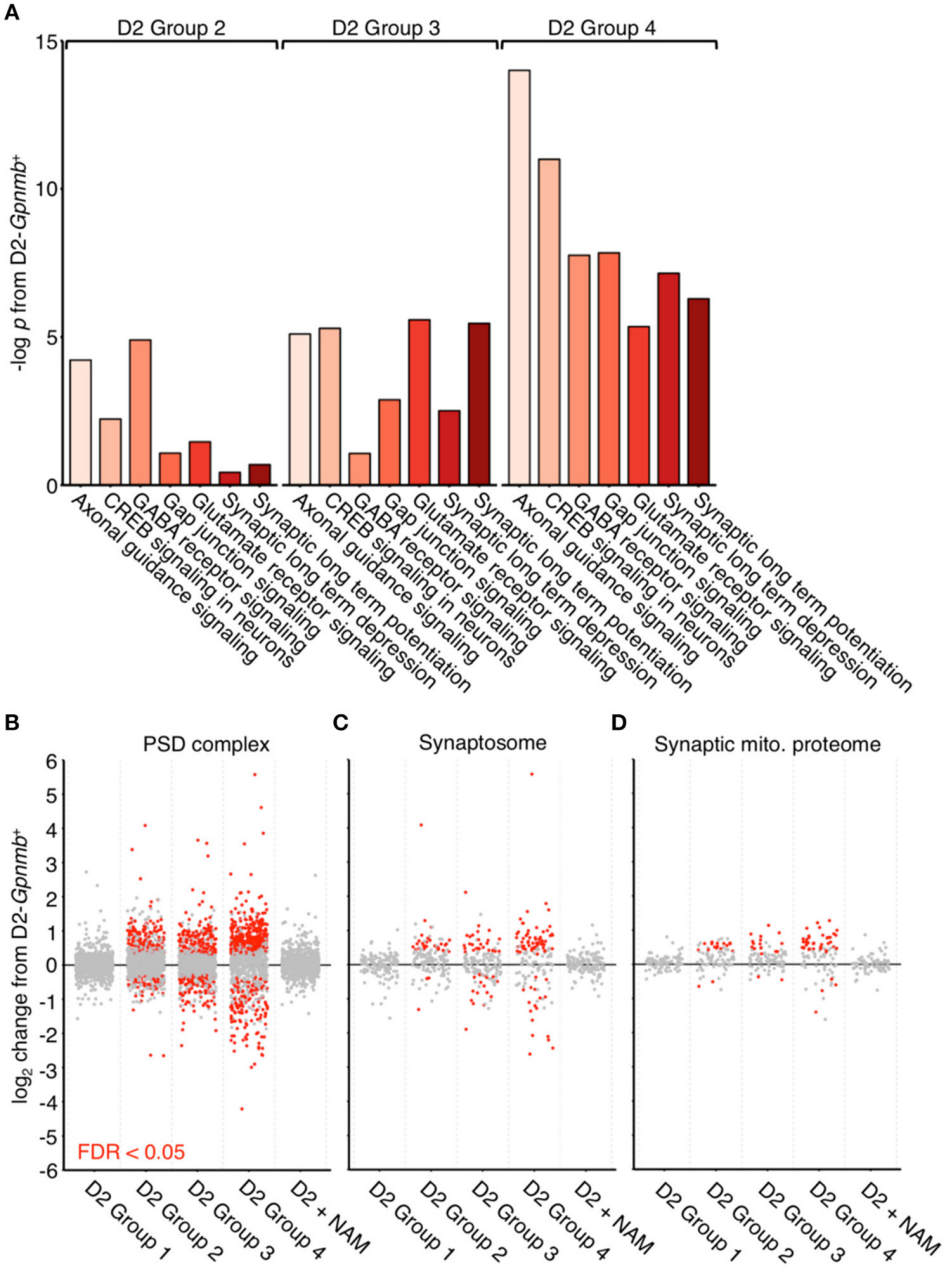
**Dysregulation of synaptic and axonal transcripts occurs early in glaucoma. (A)** Ingenuity pathway analysis of RNA transcripts from retinal ganglion cells from 9 mo D2 eyes compared to no glaucoma, age-, and sex-matched D2-*Gpnmb*^+^ controls. Pathway analysis identifies highly significant early transcriptomic changes to synaptic and axonal pathways, which become more enriched with increasing disease progression at a transcriptomic level (all samples came from mice with no glaucoma, thus these changes occur before detectable neurodegeneration). These pathways contain differentially expressed genes encoding multiple proteins that are known to be associated axon and synapse dysfunction or degeneration in other neurodegenerations. These include multiple members of the ephrin family of receptors (*Epha2, Epha4, Epha5, Epha6, Ephb2, Ephb3, Ephb4 Ephb6*; Chen et al., 2012), metabotropic glutamate receptors (*Grm3, Grm5, Grm6, Grm7*; Ribeiro et al., 2017), *Ryr3* (Balschun et al., 1999; Del Prete et al., 2014), and semaphorins (*Sema3a, Sema3b, Sema3d, Sema4a, Sema5a, Sema6a, Sema6c, Sema7a*; Shirvan et al., 2002; Good et al., 2004; Pasterkamp and Giger, 2009; Smith et al., 2015; Gutiérrez-Franco et al., 2016). There are no significantly enriched pathways in D2 Group 1 or D2 + NAM samples compared to controls. **(B–D)** Individual gene expression plots show gene sets representing proteins in the post-synaptic density **(B)**, the synaptosome **(C)**, and the synaptic mitochondrial proteome **(D)** as previously defined (Collins et al., 2006). Dots represent individual genes, gray, not differentially expressed; red, differentially expressed [at FDR (*q*) < 0.05 compared to D2-*Gpnmb*^+^ control]. For all gene sets, number of differentially expressed genes increases with increased disease progression, this implies early transcriptomic dysfunction within synaptic compartments. These differentially expressed gene changes are absent from D2 Group 1 (i.e., no glaucoma at transcriptomic level), as well as from D2 + NAM samples, suggesting that NAM prevents early synaptic transcriptomic changes.

We have recently reported metabolic and mitochondrial dysfunction in retinal ganglion cells early in glaucoma. In the current study, we reexamined publicly available RNA-seq datasets generated by our lab (Williams et al., 2017). RNA-seq was performed on RNA from retinal ganglion cells from 9 month old D2 and D2-*Gpnmb*^+^ eyes (a strain matched control that does not develop glaucoma). At this 9 mo time point, the majority of eyes have had ongoing high IOP, but degeneration of the optic nerve has not yet occurred. We used unsupervised hierarchical clustering to generate novel clusters based on global gene expression patterns separating samples into four groups: D2 Group 1 (containing the subset of D2 samples that have not yet developed any glaucoma), and D2 Groups 2, 3, and 4 [all at early molecular stages of glaucoma with increasing group number reflecting increased glaucoma progression at a transcriptomic level (Williams et al., 2017)]. Here, we analyzed these groups paying particular attention to pathways that might give clues about damaging mechanisms insulting individual neuronal compartments, especially the axon and synapse, which are both directly insulted and undergo early damage. We discovered early transcriptional dysregulation of synaptic and axonal pathways that increased in significance with disease progression (Figure 1A). We noted significant enrichment of genes within pathways corresponding to “Axon guidance signaling” (gene families including ephrins and semaphorins that have roles in synapse formation, neuronal morphogenesis, and neuronal plasticity) and “CREB signaling in neurons” (gene families that are strongly implicated in neuronal survival and synaptic plasticity), as well as other synaptic pathways (“GABA receptor signaling”, “Glutamate receptor signaling”, “Synaptic long-term depression,” and “Synaptic long-term potentiation”). Genes of interest are listed further in the figure legend for Figure 1.

### Nicotinamide supplementation prevents synapse related transcriptomic changes

A diet supplemented in NAM potently inhibits mitochondrial dysfunction during glaucoma pathogenesis (Williams et al., 2017). Analyzing our RNA-seq dataset, we discovered that retinal ganglion cells from NAM treated mice (D2 + NAM) were robustly protected from the transcriptomic dysregulation of synaptic genes that was present in untreated D2s (Figure 1B). In addition, there were no enriched pathways in samples from NAM treated D2 mice compared to D2-*Gpnmb*^+^ controls. Thus, NAM supplementation prevents the earliest transcriptomic changes relevant to synapses and axons in glaucoma.

### WLD^*S*^ and NAM increase retinal NAD levels and prevent mitochondrial changes

**Figure 2:**
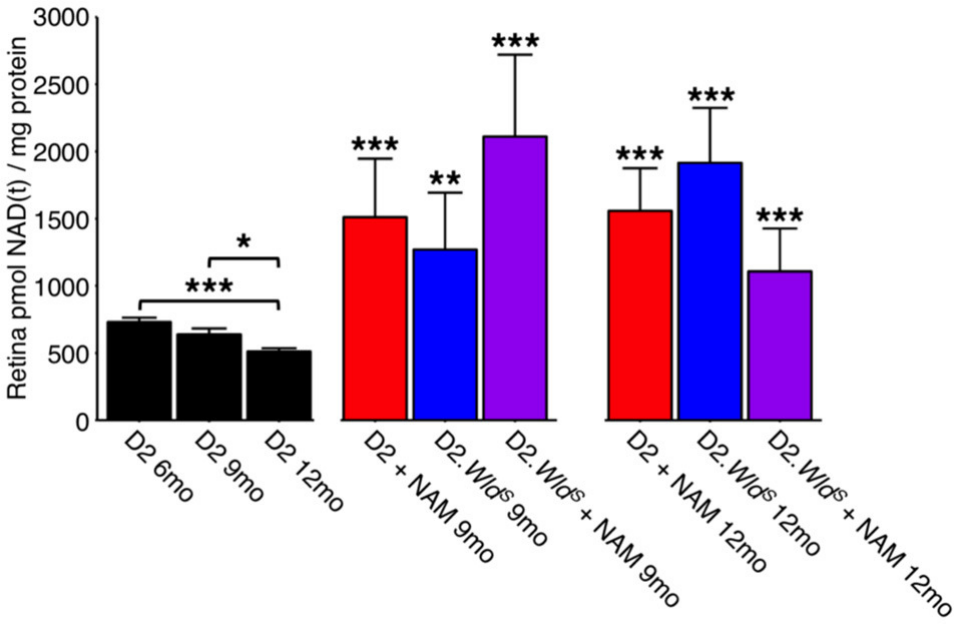
**NAM supplementation and/or WLD^**S**^ robustly restores NAD(t) levels through to 12 months**. NAD(t) levels decrease with age in D2 retinas. The presence of the *Wld*^*S*^ allele or a diet supplemented in NAM robustly increases NAD(t) levels through to 12 months (a typical end-time in this model where the majority of eyes have severe glaucoma; *n* = 22/group). ^*^P < 0.05, ^**^P < 0.01, ^***^P < 0.001; *Student's t-*test compared to age-matched D2 retinas.

**Figure 3:**
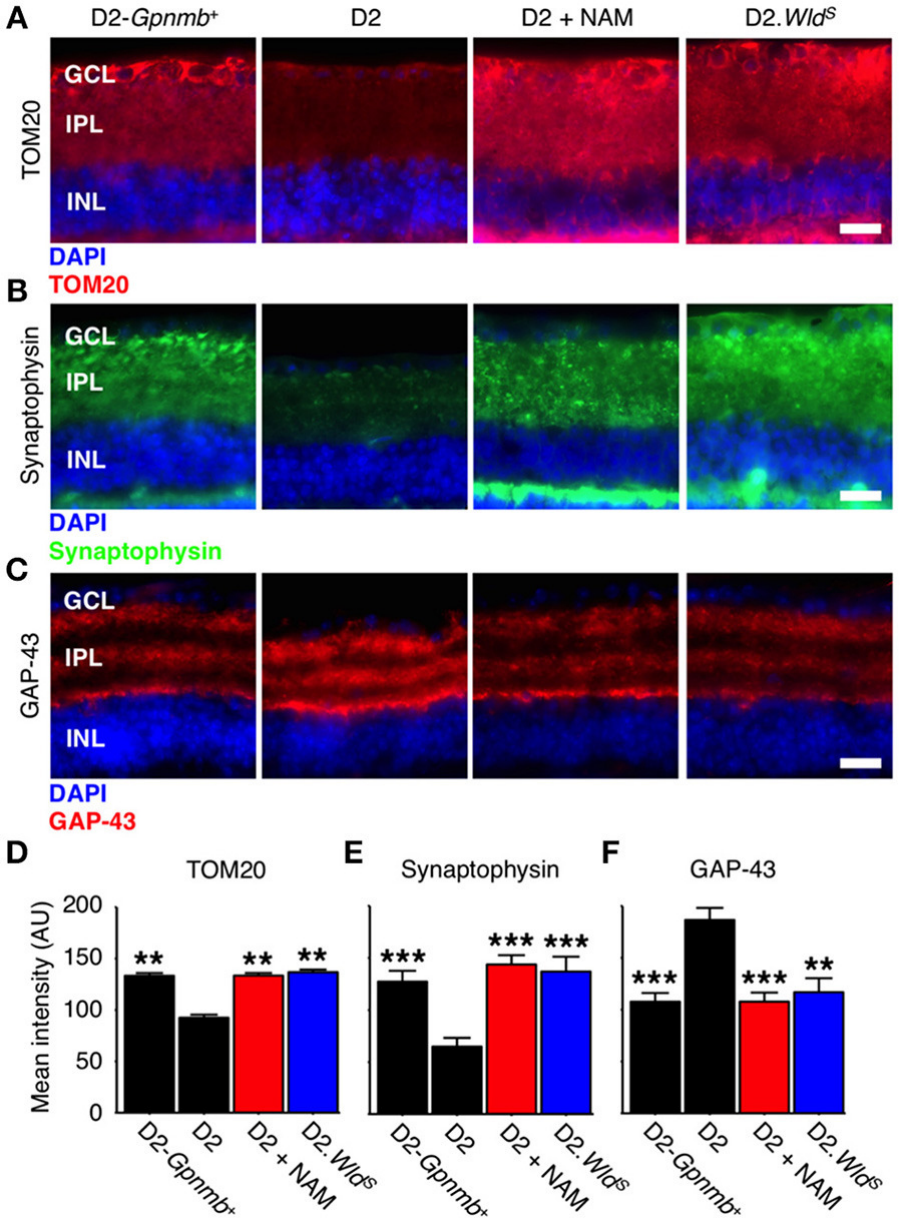
**NAM supplementation or WLD^**S**^ robustly protects from synapse elimination and neuronal stress. (A)** Presence of the *Wld*^*S*^ allele or NAM prevents decreases in TOM20 staining (red). TOM20 is a central component of an essential complex responsible for recognition and translocation of mitochondrial preproteins (Neupert, 1997). We have previously demonstrated decreased mtRNA content in glaucomatous RGCs (Williams et al., 2017). These changes are correlated with a decrease TOM20 staining within the dendrite/synapses of D2 retinas. Retinas from NAM supplemented mice or mice carrying the *Wld*^*S*^ allele have TOM20 immunoreactivity levels similar to non-glaucomatous controls. 12 mo shown for all. Quantitative data are presented in **(D)**. **(B)** Retinal ganglion cell synapses elimination begins early in glaucoma and progresses to widespread synapse loss by 12 mo. Presence of the *Wld*^*S*^ allele or NAM protects retinal ganglion cell synapses (as assessed by synaptophysin staining, *green*), even at late time points when degeneration is almost complete in untreated D2 eyes. 12 mo shown for all. Quantitative data are presented in **(E)**. **(C)** GAP-43 levels increase in the retinal ganglion cell following axon injury at the proximal end of the axon (i.e., close to the optic nerve head; Doster et al., 1991). Presence of the *Wld*^*S*^ allele or NAM prevents increases in GAP-43 (red), reflecting dampened axon injury. Quantitative data are presented in **(F)**. Images are shown from representative samples corresponding to nerve grades at 12 mo. *n* = 8/group. ^**^P < 0.01, ^***^P < 0.001; *Student's t-*test compared to D2. Scale bars = 20 μm. GCL, ganglion cell layer; IPL, inner plexiform layer; INL, inner nuclear layer.

WLD^S^ prevents axon and synapse degeneration in various models of disease. Due to its NMNAT activity, we hypothesized that WLD^S^ would increase available levels of NAD(t), in the retina similar to NAM treatment. Thus, we performed NAD [NAD(t); NAD^+^ and NADH)] assays on D2.*Wld*^*S*^ mice at two different ages, both pertinent to neurodegeneration in D2 glaucoma. WLD^S^ dramatically increased retinal NAD(t) levels throughout our experimental time points at 9 mo and at 12 mo (12 mo is a typical terminal time-point for this model, where a majority of eyes have severe neurodegeneration; Figure 2). Another effect of NAM treatment in glaucoma is to prevent mitochondrial changes (Williams et al., 2017). An early marker of mitochondrial change in glaucoma is a decrease in TOM20 immunoreactivity levels. Both NAM treatment and *Wld*^*S*^ prevented the decrease in mitochondrial TOM20 immunoreactivity levels observed in D2 glaucoma (Figures 3A,D).

### WLD^*S*^ and NAM protect from synapse elimination in glaucoma

In D2 glaucoma there is a loss of synapses which is first evident (though mild) at 9 mo of age and progresses throughout the disease (Williams et al., 2016). We tested whether NAM or WLD^S^ could protect from synapse loss out to an end stage, late-time point in this glaucoma (12 months of age where the majority of eyes have severe neurodegeneration). NAM and WLD^S^ robustly protected against retinal ganglion cell synapse loss and neuronal stress in this model (Figures 3B,C,E,F).

### WLD^S^ and NAM robustly protect from axon degeneration in glaucoma

**Figure 4:**
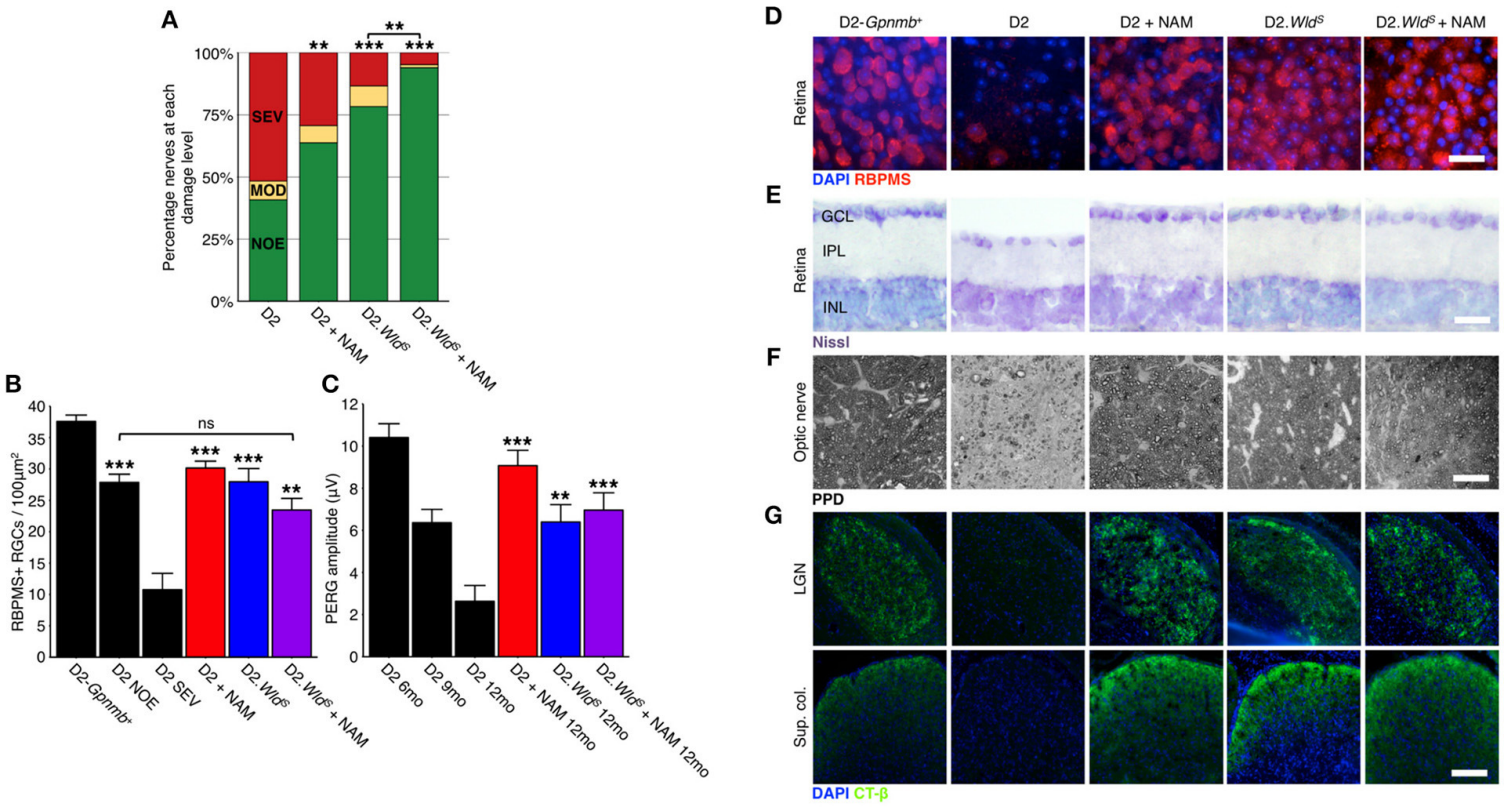
**NAM supplementation in combination with WLD^**S**^ robustly protects from glaucomatous neurodegeneration. (A)** Presence of the *Wld*^*S*^ allele or NAM significantly protect D2 eyes from optic nerve degeneration as assessed by PPD staining (examples in **F**). Combination of the *Wld*^*S*^ allele and NAM significantly protects a greater proportion of optic nerves than either treatment alone (94% of eyes have no detectable glaucoma). Green, NOE; no or early glaucoma, yellow, MOD; moderate glaucoma, red, SEV; severe glaucoma. *n* > 60/group. **(B)** Presence of the *Wld*^*S*^ allele and/or NAM protects from retinal ganglion cell soma loss **(D)**, red, RBPMS, a specific marker of retinal ganglion cells, *n* > 6/group. The density drop between D2 NOE and D2-*Gpnmb*^+^ is due to pressure induced stretching of the retina, inner plexiform layer thinning and nerve fiber layer thinning (examples in **E**). **(C)** Presence of the *Wld*^*S*^ allele or NAM significantly protect from a loss of visual function as assessed by pattern electroretinography (PERG, *n* > 20/group). PERG is a sensitive measure of retinal ganglion cell function, and thus the *Wld*^*S*^ allele and/or NAM protect even the earliest changes in glaucoma. ^***^P < 0.001 between 12 mo treatment groups and D2 12 mo. (**G**) Presence of the *Wld*^*S*^ allele and/or NAM protects retinal ganglion cells from axon loss. These axons terminate in key brain regions and have functional axoplasmic transport as assessed by CT-β labeling (*green, n* = 10/group). Retinal ganglion cell counts and axoplasmic transport images are from representative samples corresponding to NOE nerve grades at 12 mo. Representative images are shown in **D–G**. **(C,D)** ^**^P < 0.01, ^***^P < 0.001, ns, not significant. Scale bars = 20 μm **(D–F)**, 200 μm **(G)**. GCL, ganglion cell layer; IPL, inner plexiform layer; INL, inner nuclear layer.

**Figure 5:**
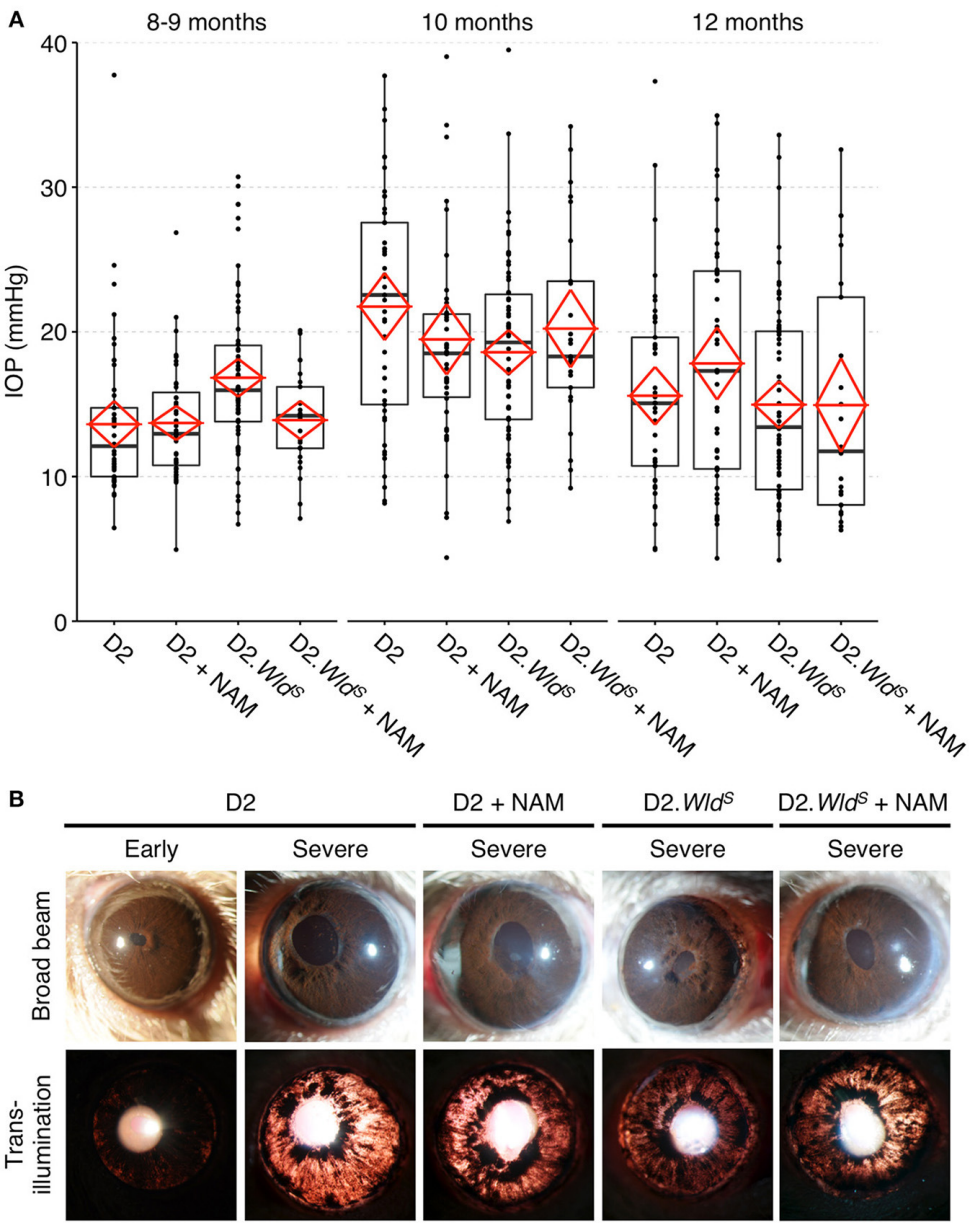
**Presence of the ***Wld***^**S**^ allele or a diet supplemented in NAM do not change clinical disease progression/presentation in treated eyes**. IOP profiles **(A)** and clinical presentation of iris disease **(B)**. IOP is not significantly different between cohorts within the same age-group. Iris disease (iris pigment dispersion resulting in asynchronous ocular hypertension) progressed at a similar rate and reached a severe state in all groups within the same time-frame. For boxplots, the upper and lower hinges represent the upper and lower quartiles. The centerline of each diamond (red) represents the mean, and the upper and lower diamond points represent 95% confidence intervals of the mean (*n* > 45 for 8–9 and 10 month groups, *n* > 25 for 12 month groups).

To test whether the levels of available enzyme and precursor (NAM) limited optic nerve protection in glaucoma, we administered NAM to D2.*Wld*^*S*^ mice (D2.*Wld*^*S*^ + NAM). D2.*Wld*^*S*^ + NAM mice had a profoundly decreased risk of developing glaucomatous neurodegeneration. NAM or WLD^S^ alone protected ~60–75% of optic nerves from degeneration, while combining NAM with WLD^S^ protected 94% (Figures 4A,F). Remarkably, this combined protection affords an ~12-fold decrease in risk of developing glaucomatous nerve damage. NAM and WLD^S^ robustly protected against retinal ganglion cell soma loss and retinal thinning (Figures 4B,D,E). Given this and along with the profound degree of optic nerve protection, the combinatorial treatment is much more effective overall. NAM and WLD^S^ protected anterograde axoplasmic transport to major retinal ganglion cell targets in the brain (Figure 4G), and electrophysiological function of retinal ganglion cells (as assessed by pattern electroretinography) to levels similar to controls (Figure 4C). Importantly, neither NAM nor WLD^S^ changed the clinical presentation of the disease (iris disease or increased IOP; Figure 5).

As individually protected eyes (NAM or WLD^S^ alone) are already indistinguishable from controls, a greater degree of protection is not expected within eyes for the combinatorial treatment (D2.*Wld*^*S*^ + NAM). However, as the combined treatment protects a significantly greater proportion of optic nerves as well as that other retinal ganglion cell parameters are also protected in eyes with protected nerves, these data indicate that in D2.*Wld*^*S*^ + NAM eyes retinal ganglion cells and axonal transport are protected in a greater percentage of eyes. Thus, the benefit of WLD^S^ + NAM is in that it fully prevents degeneration in all retinal ganglion cell compartments tested in the presence of elevated IOP in a greater percentage of eyes than either condition alone.

## Discussion

Neurons exist on a metabolic knife-edge primarily reliant on mitochondrial oxidative respiration and NAD dependent metabolism to meet energy demands. Our previous studies have demonstrated that NAD(t) depleting processes contribute to glaucoma (Williams et al., 2017). Here, we find that dietary supplementation of NAM (an NAD precursor) in combination with WLD^S^ protects D2 mice from glaucoma (preventing functional vision loss, retinal, and optic nerve damage). Importantly, the combination of WLD^S^ and NAM protects many more eyes (94% not developing glaucoma) than either treatment alone.

We previously determined that an age-dependent decline of NAD(t) renders retinal ganglion cells vulnerable to insults from elevated IOP, leading to subsequent glaucomatous degeneration (Williams et al., 2017). A diet supplemented with NAM boosts NAD(t) levels and reduces susceptibility to glaucoma. To better understand the effects of NAM and importance of NAD(t) levels, we studied the effects of NAM and an NAD-producing enzyme, WLD^S^, on pre-degenerative changes in glaucoma. NAM treatment prevented synaptic and mitochondrial changes based on transcript and protein expression data. WLD^S^ is well-established to delay axon degeneration and provide variable, context-dependent protection of soma or synapses (Deckwerth and Johnson, 1994; Gillingwater et al., 2006; Beirowski et al., 2008). Here we show that WLD^S^ increases NAD(t) levels in the retina, and in addition to preventing optic nerve and soma degeneration, WLD^S^ prevents pre-degenerative changes in glaucoma (including synaptic loss, and a decrease in mitochondrial TOM20 levels) similar to NAM treatment.

As both NAM and WLD^S^ independently boost retinal NAD(t) levels and have similar protective effects in glaucoma, these data are consistent with WLD^S^ protecting retinal ganglion cells by increasing NAD(t) levels. They provide further support to the concept that maintaining NAD(t) levels is critical in enabling retinal ganglion cells to resist changes caused by glaucomatous insults. When presented in combination, D2.*Wld*^*S*^ and NAM act together to protect significantly more optic nerves from glaucoma than when tested alone. The combined treatment lowers the risk of developing glaucoma by ~12-fold in this model. The combined treatment did not further increase NAD(t), however, possibly due to a physiological limit of NAD(t) production within the retina (the activity of NAMPT, the rate limiting enzyme in NAM salvage, may be limiting). Nevertheless, it remains possible that the combined treatment is more protective as it enhances the supply of NAD(t) without further increasing its steady state level (i.e., greater NAD flux). Alternatively NAM and WLD^S^ may act via different mechanisms. It is conceivable that NAM administration directly protects retinal mitochondria (possibly neuronal and non-neuronal) from dysfunction through its NAD dependent actions on the sirtuin family of enzymes while WLD^S^ protects axons. NAM may also protect by modulating DNA damage responses and/or vascular tone, due to its direct inhibitory effects on PARP and cyclic ADP ribose (Sethi et al., 1996; Geiger et al., 2000; Gibson and Kraus, 2012). WLD^S^ also has NAD independent roles in protecting axons (Zhai et al., 2006, 2008; Coleman and Freeman, 2010; Antenor-Dorsey and O'Malley, 2012; Kitay et al., 2013; O'Donnell et al., 2014). WLD^S^ has been shown to partially attenuate mitochondrial transport defects following axotomy (O'Donnell et al., 2013), and our TOM20 results may be in part due to increased mitochondrial motility following induction of elevated IOP. Further studies are required to fully elucidate the mechanism of NAM and WLD^S^ protection.

Our group has previously demonstrated that overexpression of murine *Nmnat1* also protects from glaucoma (Williams et al., 2017). A benefit of WLD^S^ over NMNAT1 alone is suggested to be the localization of WLD^S^ at the site of injury, likely the axon or synapse (Beirowski et al., 2009), whereas NMNAT1 is predominantly nuclear. Consistent with this hypothesis, strategies that drive misexpression of NMNAT1 outside of the nucleus result in robust axonal protection comparable to that of WLD^S^ (Sasaki et al., 2009). Here, we show that the combination of WLD^S^ and NAM is profoundly protective with 94% of treated eyes not developing glaucoma. Previously, we observed protection in only 84% of NAM + *Nmnat1* treated eyes (using the same NAM dose as the current study). With the caveats that its gene was inherited and not virally transduced (unlike *Nmnat1*) and *in vivo* NMNAT1 activity levels are difficult to compare, WLD^S^ may provide a more effective therapy (*Fisher's exact* test, *P* < 0.01, NAM + WLD^S^ vs. NAM + *Nnmat1*). However, future experiments are needed to definitively test this. The axonal protection conferred by the murine *Wld*^*S*^ allele is conserved across species, delaying axon degeneration in mice (Lunn et al., 1989), rats (Adalbert et al., 2005; Beirowski et al., 2008), zebrafish (O'Donnell et al., 2014), and flies (Hoopfer et al., 2006), as well as human neuronal cultures (Kitay et al., 2013). Although significant work is required before moving *Wld*^*S*^ into the clinic, based on these collective data there is definite promise for axon and synapse targeted treatments that manipulate molecules in the NAD pathway.

The combination of NAM and WLD^S^ offers a potent neuroprotective strategy for glaucoma, and is likely to benefit even patients that are refractory to IOP lowering treatments. Although glaucoma may be uniquely sensitive to WLD^S^ mediated protection, our current data raise the possibility of combining *Wld*^*S*^ (and/or *Nmnat1*) gene therapy with NAM administration in other neurodegenerative conditions. Such a combination has great potential to provide more effective protection than *Wld*^*S*^ alone. It is likely to be of especial value for age-related axonopathies. Importantly, this combination may even extend the protection beyond the axon to other neuronal compartments providing much-improved outcomes.

## Author contributions

PW and JH: designed and performed experiments, wrote the manuscript; NF, BC, and KC: performed experiments; SJ: conceived experiments, wrote the manuscript.

## Funding

The Jackson Laboratory Fellowships (PW, JH), NEI NIH (R01-EY11721) (SJ), the Barbara and Joseph Cohen foundation, the Partridge Foundation, and the Lano Family Foundation (SJ). SJ is an Investigator of HHMI.

### Conflict of interest statement

The authors declare that the research was conducted in the absence of any commercial or financial relationships that could be construed as a potential conflict of interest.

## Abbreviations

FDR: false discovery rate
GCL: ganglion cell layer
INL: inner nuclear layer
IPL: inner plexiform layer
IOP: intraocular pressure
mo: months of age
NAD: nicotinamide adenine dinucleotide
NAM: nicotinamide
NMN: nicotinamide mononucleotide
OCT: optimal cutting temperature
ON: overnight
RNA-seq: RNA-sequencing
RT: room temperature
*Wld* ^*S*^: Wallerian degeneration slow allele.
